# Supplementary material for: A Likelihood Approach for Real-Time Calibration of Stochastic Compartmental Epidemic Models
Source: PLoS Comput Biol. 2017 Jan 17;13(1):e1005257. doi: 10.1371/journal.pcbi.1005257 (PMC5240920; doi:10.1371/journal.pcbi.1005257)
Supplement: S1 File — (TAR.GZ) [file pcbi.1005257.s014.tar.gz › HSPH_Online-SI-Revision/MSS10/n100-extreme/n100-extreme_NEW_table-4.pdf]

1	R0	{2.76052, 16.3913, 0.1}	{1.48053, 4.30277, 0.06}	{{1.44762, 4.00571, 0.}, {1.44762, 4.00571, 0.}}	{46.3677, 73.7496, 154.}	inf	10419.	2701.13	{2300.36, 2765.62}	74.0749	0.601929	newly	5804.	5678.54	{5167., 6117.}	5.19263	0.0231762	Reff	2.28524	1.33804	{1.3103, 1.31914}	41.4488	ar	92090.	55786.6	{53758., 56063.}	39.4217
2	R0	{2.18202, 15.1734, 3.}	{1.25301, 2.62305, 0.07}	{{1.24751, 2.5364, 0.}, {1.24751, 2.5364, 0.}}	{42.5755, 82.7128, 97.6667}	inf	10970.	1855.07	{1533.56, 2057.25}	83.0896	0.778259	newly	5914.	5337.64	{4610., 6060.}	10.9229	0.0518338	Reff	1.6841	1.0812	{1.07413, 1.08088}	35.7998	ar	84264.	37003.4	{35137., 37963.}	56.0863
3	R0	{2.57891, 12.5971, 1.}	{1.48145, 3.92522, 0.}	{{1.47488, 3.83841, 0.}, {1.47608, 4.06661, 0.}}	{42.555, 68.8403, 100.}	inf	8525.	2529.8	{2241.93, 2788.61}	70.3249	0.529849	newly	6571.	6051.27	{5516., 6562.}	8.57115	0.0396593	Reff	2.202	1.35369	{1.34476, 1.35343}	38.5247	ar	90302.	56891.2	{55865., 57442.}	36.9989
4	R0	{2.5856, 9.28508, 0.1}	{2.85003, 10.2178, 0.04}	{{2.91001, 10.1535, 0.}, {2.91001, 10.5991, 0.}}	{12.9509, 15.2558, 132.}	inf	2564.	2738.32	{2617.93, 2997.78}	12.1861	0.0534219	newly	3492.	3505.18	{3282., 3781.}	4.58076	0.01975	Reff	2.47933	2.72725	{2.77446, 2.7884}	12.6161	ar	90312.	92540.6	{93107., 93608.}	3.83761
5	R0	{2.4406, 12.1341, 1.}	{2.42826, 12.3674, 1.5}	{{1.74092, 5.83416, 0.}, {2.688, 14.8279, 2.}}	{15.3055, 30.4903, 98.}	inf	4472.	4393.57	{2138.04, 5335.59}	26.052	0.134216	newly	3644.	3604.57	{3384., 4002.}	5.36142	0.0231807	Reff	2.2517	2.23641	{1.64469, 2.46325}	14.1817	ar	88597.	85703.1	{70755., 91590.}	7.98385
6	R0	{2.72155, 15.4094, 2.}	{2.10687, 10.0443, 0.2}	{{1.93419, 8.56054, 0.}, {2.93371, 17.1903, 1.}}	{25.392, 38.9774, 90.}	inf	8392.	5457.6	{4351.53, 9737.36}	40.946	0.234711	newly	5118.	5062.92	{4818., 5339.}	3.46463	0.0152007	Reff	2.34317	1.86778	{1.73529, 2.48398}	22.4666	ar	91682.	80268.5	{77358., 93625.}	13.2059
7	R0	{2.63781, 14.3935, 0.1}	{1.4847, 4.01583, 0.06}	{{1.47488, 3.83841, 0.}, {1.47608, 4.06661, 0.}}	{43.7148, 72.0997, 154.}	inf	9079.	2639.6	{2345.6, 2893.04}	70.9264	0.539642	newly	5875.	6119.89	{5535., 6774.}	7.12357	0.0298458	Reff	2.22919	1.35016	{1.33785, 1.34763}	39.4326	ar	91192.	57159.7	{55752., 57695.}	37.3194
8	R0	{2.82391, 16.104, 1.}	{1.68654, 6.34789, 0.66}	{{1.44762, 4.00571, 0.}, {2.00349, 9.42599, 2.}}	{40.381, 60.9856, 86.}	inf	8948.	3343.31	{1969.89, 4940.69}	62.7164	0.466891	newly	5170.	4955.88	{4541., 5435.}	6.69246	0.0301806	Reff	2.41478	1.53223	{1.33374, 1.7884}	36.6437	ar	92664.	65319.3	{54056., 79135.}	29.5341
9	R0	{2.80645, 14.6457, 0.1}	{1.47781, 4.06665, 0.04}	{{1.44762, 3.83841, 0.}, {1.47608, 4.06661, 0.}}	{47.3423, 72.2331, 136.}	inf	5809.	1532.28	{1321.83, 1725.81}	73.6223	0.582313	newly	4055.	3727.03	{3232., 4279.}	10.6128	0.0493054	Reff	2.54301	1.40222	{1.37566, 1.40309}	44.8597	ar	92567.	56713.6	{54684., 57527.}	38.7324
10	R0	{1.86213, 5.4271, 0.1}	{2.35254, 9.20828, 0.87}	{{1.81768, 5.68021, 0.}, {2.79531, 12.1249, 2.}}	{27.5793, 70.0861, 860.}	inf	1977.	3368.44	{1981.61, 4511.56}	71.6359	0.221526	newly	4393.	4072.37	{3752., 4427.}	7.87913	0.0361125	Reff	1.77612	2.20829	{1.73301, 2.59841}	25.5668	ar	75362.	85682.	{73710., 92524.}	14.8408
11	R0	{2.27349, 14.6338, 1.}	{1.24893, 2.5536, 0.04}	{{1.24751, 2.5364, 0.}, {1.24751, 2.5364, 0.}}	{45.0657, 82.55, 102.}	inf	10491.	1801.43	{1493.32, 2056.5}	82.8288	0.769536	newly	6044.	5494.08	{4575., 6309.}	11.2948	0.0537406	Reff	1.80677	1.10104	{1.09663, 1.10366}	39.0601	ar	85805.	37077.	{35444., 38600.}	56.7892
12	R0	{2.77261, 14.1894, 0.1}	{1.7227, 5.69529, 0.14}	{{1.74092, 5.83416, 0.}, {1.75359, 5.92488, 1.}}	{37.8673, 59.8624, 212.}	inf	6492.	2633.28	{2288.27, 2857.96}	59.438	0.394957	newly	4508.	4687.13	{4349., 4949.}	5.85248	0.0248401	Reff	2.48165	1.60822	{1.6202, 1.63346}	35.1956	ar	92508.	69890.2	{69879., 71517.}	24.4496
13	R0	{2.65402, 19.3935, 2.}	{1.77524, 9.03178, 1.02}	{{1.77826, 9.06807, 1.}, {1.77826, 9.06807, 1.}}	{33.1113, 53.4288, 50.}	inf	11250.	5160.88	{4983.6, 5351.91}	54.1255	0.338996	newly	4926.	4887.68	{4726., 5056.}	2.19285	0.00959939	Reff	2.14402	1.54215	{1.54139, 1.54759}	28.0723	ar	91184.	72140.9	{71780., 72712.}	20.8842
14	R0	{1.75297, 4.45354, 0.1}	{1.40839, 2.04972, 0.13}	{{1.39162, 1.89883, 0.}, {1.43965, 2.60601, 1.}}	{19.6572, 53.9754, 204.}	inf	699.	377.025	{156.685, 608.465}	46.6315	0.343759	newly	1855.	2665.71	{1048., 4266.}	64.9666	0.23913	Reff	1.72278	1.38865	{1.36892, 1.41938}	19.3951	ar	71263.	51495.9	{49518., 54083.}	27.7382
15	R0	{1.99928, 7.1501, 0.1}	{1.95369, 7.2843, 0.04}	{{1.737, 5.43578, 0.}, {2.22376, 9.35432, 0.}}	{13.966, 28.9326, 136.}	inf	3389.	3223.88	{2201.61, 4346.57}	29.0063	0.135384	newly	4922.	4569.64	{4176., 4841.}	7.44779	0.0342702	Reff	1.85853	1.81636	{1.62905, 2.05393}	12.931	ar	79602.	76423.	{70267., 85182.}	10.4636
16	R0	{2.67398, 10.3134, 1.}	{2.00665, 6.24185, 0.33}	{{1.98388, 6.25659, 0.}, {2.14326, 6.48739, 2.}}	{24.9566, 39.4783, 101.}	inf	4133.	2456.22	{2183.6, 2815.63}	40.5705	0.235132	newly	4823.	4632.01	{4173., 5106.}	6.76363	0.0305234	Reff	2.49563	1.90573	{1.88166, 2.03557}	23.637	ar	91289.	79129.3	{78811., 83437.}	13.3201
17	R0	{2.44634, 16.3226, 0.1}	{1.24751, 2.5364, 0.}	{{1.24751, 2.5364, 0.}, {1.24751, 2.5364, 0.}}	{49.0049, 84.4608, 100.}	inf	10530.	1487.27	{1190.7, 1784.42}	85.8759	0.854966	newly	5419.	4693.95	{3744., 5554.}	14.9989	0.0736438	Reff	1.9738	1.11935	{1.11546, 1.12294}	43.2896	ar	88710.	36518.7	{34786., 38079.}	58.8336
18	R0	{2.02168, 10.1676, 0.1}	{1.9396, 9.61721, 1.18}	{{1.95791, 9.82151, 1.}, {1.95791, 9.82151, 1.}}	{4.05986, 5.41314, 1080.}	inf	4597.	4355.93	{4173.73, 4558.99}	5.31962	0.024823	newly	4153.	4157.83	{4035., 4295.}	2.0561	0.00891926	Reff	1.82331	1.7538	{1.76474, 1.77089}	3.8122	ar	80299.	77810.8	{77731., 78866.}	3.09866
19	R0	{2.89969, 15.009, 0.1}	{1.75372, 5.91799, 0.12}	{{1.737, 5.43578, 0.}, {1.75359, 5.92488, 1.}}	{39.5204, 60.5704, 196.}	inf	6390.	2645.39	{2266.8, 2787.04}	58.6011	0.392452	newly	4254.	4573.99	{4257., 4857.}	7.90197	0.032604	Reff	2.60468	1.63988	{1.62475, 1.64055}	37.0407	ar	93349.	70650.	{70009., 71584.}	24.3162
20	R0	{2.68933, 7.34928, 0.1}	{1.81911, 4.12803, 0.13}	{{1.65456, 3.37285, 0.}, {1.94705, 4.66877, 1.}}	{32.3583, 43.8308, 206.}	inf	478.	295.924	{160.316, 425.278}	38.1899	0.243175	newly	1059.	1071.69	{633., 1470.}	25.3513	0.118847	Reff	2.66728	1.8074	{1.64327, 1.93501}	32.2379	ar	91574.	72848.9	{66484., 78423.}	20.448
21	R0	{2.15352, 9.28074, 0.1}	{1.69737, 5.29193, 0.04}	{{1.53501, 4.02511, 0.}, {1.74592, 5.83416, 0.}}	{21.6164, 43.7779, 132.}	inf	3961.	2254.06	{1678.2, 2580.21}	43.6129	0.25596	newly	4282.	4439.49	{3965., 4817.}	6.93438	0.0292916	Reff	1.99267	1.59895	{1.45328, 1.64497}	20.1686	ar	83845.	68236.	{59994., 71361.}	18.806
22	R0	{2.21953, 9.04387, 0.1}	{2.29928, 9.53609, 0.9}	{{1.53501, 4.02511, 0.}, {2.68687, 12.2271, 2.}}	{21.2592, 37.3713, 868.}	inf	3707.	3927.68	{1612.62, 5218.82}	37.7598	0.178891	newly	4322.	4364.03	{4005., 4637.}	5.21078	0.0226508	Reff	2.06581	2.12792	{1.46084, 2.46522}	19.7599	ar	84856.	82215.4	{60434., 91584.}	12.4719
23	R0	{2.21987, 14.5347, 1.}	{1.45493, 5.15973, 2.8}	{{1.38889, 4.24222, 3.}, {1.47651, 5.4396, 3.}}	{34.4588, 64.5006, 196.}	inf	10313.	3645.94	{2887.42, 4036.45}	64.6471	0.459486	newly	5905.	5757.58	{5443., 6075.}	3.97934	0.0177163	Reff	1.76493	1.2534	{1.20894, 1.27237}	28.9828	ar	85067.	55004.6	{49852., 57519.}	35.3396
24	R0	{2.84268, 17.9659, 1.}	{1.25952, 2.62456, 0.}	{{1.24751, 2.5364, 0.}, {1.24751, 2.5364, 0.}}	{55.6925, 85.3914, 100.}	inf	10277.	1471.31	{1138.26, 1754.74}	85.6835	0.853379	newly	5220.	4700.59	{3797., 5559.}	13.1584	0.0638125	Reff	2.35894	1.15598	{1.14209, 1.15103}	50.9956	ar	92836.	38284.	{36019., 38852.}	58.7617
25	R0	{2.52706, 10.3679, 0.1}	{1.50378, 3.72646, 0.49}	{{1.37363, 2.81014, 0.}, {1.53501, 4.02511, 2.}}	{40.4931, 64.0577, 544.}	inf	4409.	1494.81	{1144.74, 1776.22}	66.0963	0.477093	newly	4655.	4239.97	{3534., 4813.}	12.1927	0.0572566	Reff	2.33579	1.43368	{1.31987, 1.46421}	38.6211	ar	89623.	58253.	{49900., 60941.}	35.0022
26	R0	{2.62835, 19.1088, 1.}	{1.38839, 4.34037, 1.16}	{{1.24751, 2.5364, 0.}, {1.85376, 9.90957, 3.}}	{47.1763, 77.286, 126.}	inf	12549.	2804.38	{1459.08, 6468.43}	77.6526	0.712299	newly	5505.	5207.38	{4532., 5765.}	8.01453	0.0368923	Reff	2.05547	1.21777	{1.10929, 1.55935}	40.7548	ar	90908.	47475.6	{36063., 75234.}	47.7762
27	R0	{2.76831, 19.4155, 2.}	{1.6745, 7.29849, 1.05}	{{1.67405, 7.28757, 1.}, {1.67405, 7.28757, 1.}}	{39.512, 62.4089, 47.5}	inf	16382.	6403.16	{6212.42, 6571.61}	60.9134	0.408082	newly	6900.	7141.29	{6909., 7347.}	3.71493	0.0157559	Reff	1.96727	1.35706	{1.35384, 1.36021}	31.0182	ar	92175.	67930.8	{67369., 68488.}	26.3024
28	R0	{2.96609, 16.8903, 1.}	{1.46154, 4.0058, 0.}	{{1.44762, 3.83841, 0.}, {1.47488, 4.06661, 0.}}	{50.7251, 76.2834, 100.}	inf	11248.	2517.36	{2274.33, 2763.69}	77.6195	0.651845	newly	6075.	5772.04	{5225., 6315.}	7.10255	0.0323764	Reff	2.44352	1.33163	{1.31641, 1.34673}	45.5039	ar	94024.	55655.	{54334., 57170.}	40.8077
29	R0	{2.17046, 14.39, 0.1}	{1.32819, 3.70776, 0.99}	{{1.24751, 2.5364, 0.}, {1.41571, 4.94504, 2.}}	{38.806, 74.2338, 994.}	inf	8850.	2198.99	{1363.25, 3009.75}	75.1527	0.628128	newly	5160.	4842.08	{4358., 5417.}	8.93876	0.0417153	Reff	1.78813	1.182	{1.11654, 1.2524}	33.8976	ar	84001.	44144.1	{35384., 52930.}	47.4481
30	R0	{2.88772, 12.4647, 1.}	{1.6059, 3.32148, 0.46}	{{1.39162, 1.89883, 0.}, {1.71582, 3.97874, 1.}}	{44.4986, 73.3529, 68.}	inf	3959.	1077.61	{467.51, 1470.41}	72.7808	0.635754	newly	3670.	4259.1	{3157., 5686.}	24.8327	0.0972779	Reff	2.71599	1.55424	{1.35296, 1.66308}	42.9142	ar	93273.	60642.2	{49546., 71250.}	35.0158
31	R0	{2.46831, 8.14308, 0.1}	{2.14565, 6.15266, 0.18}	{{1.65456, 3.37285, 0.}, {2.91001, 10.3743, 1.}}	{19.5876, 34.166, 252.}	inf	1290.	1025.46	{557.103, 1679.81}	32.1869	0.174712	newly	2181.	2327.58	{1991., 2726.}	12.6933	0.0521995	Reff	2.41433	2.10288	{1.6305, 2.83602}	19.235	ar	88762.	80594.8	{67132., 93363.}	11.1037
32	R0	{1.93236, 7.38987, 0.1}	{2.65361, 13.2532, 3.}	{{2.6536, 13.2503, 3.}, {2.6536, 13.2503, 3.}}	{37.3247, 79.3426, 2900.}	inf	2899.	5444.84	{5275.23, 5613.27}	87.8177	0.273546	newly	4137.	4184.28	{4065., 4335.}	2.22045	0.0095247	Reff	1.80908	2.41672	{2.41219, 2.42111}	33.5887	ar	77654.	91071.7	{90868., 91234.}	17.2788
33	R0	{2.82251, 17.6954, 1.}	{1.91411, 8.56876, 1.39}	{{1.44762, 4.00571, 0.}, {2.00349, 9.42599, 2.}}	{32.1842, 51.5763, 63.}	inf	8313.	4199.33	{2042.83, 4764.54}	49.4848	0.313586	newly	4307.	4596.27	{4382., 4844.}	6.87044	0.0286215	Reff	2.44362	1.73352	{1.34663, 1.81417}	29.0593	ar	92745.	75660.	{55443., 80078.}	18.4215
34	R0	{1.87083, 12.3994, 1.}	{1.29712, 4.08073, 0.25}	{{1.25474, 3.26445, 0.}, {1.3618, 4.98002, 1.}}	{30.6659, 67.0893, 77.}	inf	6523.	2056.85	{1544.48, 2516.16}	68.4677	0.507793	newly	4361.	3965.87	{3521., 4242.}	9.59344	0.0446627	Reff	1.57679	1.15105	{1.11762, 1.20282}	27.0004	ar	76282.	41895.1	{36514., 47912.}	45.0786
35	R0	{2.95779, 16.3003, 1.}	{2.40818, 11.8708, 0.31}	{{2.36114, 11.6557, 0.}, {2.44101, 12.0941, 1.}}	{18.5896, 27.1743, 75.}	inf	6128.	4350.81	{4163.48, 4553.28}	29.0012	0.150038	newly	3852.	3698.43	{3574., 3825.}	4.05322	0.0181044	Reff	2.67834	2.22314	{2.17946, 2.25534}	17.0057	ar	93873.	87842.6	{87172., 88624.}	6.42675
36	R0	{2.18718, 14.261, 3.}	{1.24751, 2.5364, 0.}	{{1.24751, 2.5364, 0.}, {1.24751, 2.5364, 0.}}	{42.9625, 82.2144, 100.}	inf	9128.	1574.12	{1287.88, 1821.57}	82.755	0.766854	newly	5307.	4918.29	{4092., 5658.}	10.7712	0.0504795	Reff	1.78461	1.11213	{1.10904, 1.11562}	37.682	ar	84379.	36512.2	{35289., 37938.}	56.7283
37	R0	{2.44912, 9.31118, 0.1}	{2.31674, 8.20111, 0.64}	{{1.4804, 3.12918, 0.}, {2.74056, 10.6768, 1.}}	{13.2346, 20.421, 618.}	inf	3260.	3065.67	{1076.47, 4024.44}	24.192	0.134196	newly	3995.	4380.87	{4068., 4737.}	11.9962	0.0493333	Reff	2.30876	2.18443	{1.43097, 2.55737}	12.5475	ar	88442.	83548.	{57588., 91820.}	8.42273
38	R0	{2.05949, 5.72514, 0.1}	{1.42368, 2.13109, 0.12}	{{1.39162, 1.89883, 0.}, {1.43965, 2.60601, 1.}}	{30.8721, 62.7766, 196.}	inf	809.	347.546	{116.883, 547.128}	57.04	0.432231	newly	2064.	2392.25	{978., 4000.}	46.9268	0.198599	Reff	2.02491	1.40631	{1.37236, 1.42397}	30.5495	ar	81587.	52578.	{49398., 55015.}	35.556
39	R0	{1.75505, 8.52404, 0.1}	{1.4902, 5.27637, 0.43}	{{1.24751, 2.5364, 0.}, {2.1245, 12.2645, 2.}}	{24.2386, 57.4319, 466.}	inf	4468.	2812.33	{1095.05, 6507.27}	57.5061	0.392072	newly	4543.	4534.55	{3610., 5238.}	9.36628	0.0418902	Reff	1.55847	1.34018	{1.14341, 1.84023}	21.9406	ar	70925.	50508.3	{35802., 82837.}	36.547
40	R0	{2.24724, 13.7861, 1.}	{1.24751, 2.5364, 0.}	{{1.24751, 2.5364, 0.}, {1.24751, 2.5364, 0.}}	{44.4869, 81.6017, 100.}	inf	10101.	1848.17	{1583.5, 2105.49}	81.7031	0.740277	newly	6145.	5623.38	{4793., 6290.}	10.2063	0.0480433	Reff	1.78404	1.09333	{1.09004, 1.09658}	38.716	ar	85257.	37172.4	{35463., 38726.}	56.3996
41	R0	{1.77635, 8.62406, 0.1}	{1.29993, 3.11428, 0.24}	{{1.24751, 2.5364, 0.}, {1.24751, 2.5364, 0.}}	{29.1494, 68.8193, 328.}	inf	5330.	1989.49	{1359.46, 1975.03}	68.3878	0.501671	newly	5334.	5149.42	{4354., 6014.}	10.7364	0.0487538	Reff	1.53452	1.1633	{1.1204, 1.12857}	26.0994	ar	72941.	39732.9	{35531., 38830.}	47.1202
42	R0	{1.80397, 5.4932, 1.}	{2.01895, 6.69668, 1.12}	{{1.43965, 2.60601, 0.}, {2.79531, 12.1249, 2.}}	{29.4547, 67.2403, 46.}	inf	2276.	2793.33	{1007.9, 5030.52}	64.1182	0.255319	newly	4341.	4886.29	{4236., 5866.}	14.3628	0.0551522	Reff	1.7106	1.89494	{1.37917, 2.57164}	27.5131	ar	73232.	72941.5	{53292., 92364.}	22.1168
43	R0	{2.83096, 16.7077, 0.1}	{1.49935, 4.49248, 0.09}	{{1.44762, 4.00571, 0.}, {1.739, 7.10524, 0.}}	{47.0373, 73.1113, 184.}	inf	8628.	2250.59	{1777.92, 3404.71}	73.9153	0.601773	newly	4809.	4664.23	{4117., 5109.}	6.46517	0.0293624	Reff	2.43862	1.38607	{1.33991, 1.58882}	43.1618	ar	92740.	56958.1	{53682., 70459.}	38.583
44	R0	{2.73678, 11.6021, 1.}	{2.65414, 11.1792, 1.7}	{{1.81768, 5.68021, 1.}, {2.79531, 12.1249, 2.}}	{6.61189, 10.9527, 84.}	inf	6552.	6182.	{3270.82, 6895.01}	9.80114	0.0539365	newly	6185.	5916.82	{5702., 6119.}	4.46209	0.0199514	Reff	2.44682	2.37902	{1.68908, 2.50126}	6.07813	ar	92003.	90009.6	{74154., 92647.}	3.03091
45	R0	{2.14235, 6.98601, 0.1}	{1.53229, 3.25391, 1.03}	{{1.43965, 2.60601, 0.}, {1.98388, 6.25659, 2.}}	{28.8218, 54.4012, 960.}	inf	2850.	1267.82	{763.732, 2250.47}	56.0926	0.389838	newly	4808.	4570.81	{3417., 5830.}	16.0035	0.0739038	Reff	2.02463	1.4713	{1.38193, 1.87925}	27.6572	ar	82974.	58404.	{52946., 78830.}	29.8054
46	R0	{2.23409, 9.21289, 0.1}	{2.75955, 12.8271, 0.}	{{2.64721, 11.9965, 0.}, {2.86001, 13.5542, 0.}}	{25.9018, 43.5393, 100.}	inf	2544.	3914.99	{3569.95, 4292.02}	58.1114	0.202696	newly	3017.	3320.77	{3190., 3469.}	10.4458	0.0429578	Reff	2.12542	2.58596	{2.48623, 2.6796}	23.9648	ar	85163.	91425.4	{90872., 93165.}	9.21941
47	R0	{2.12258, 8.15265, 0.1}	{1.58952, 4.4568, 0.01}	{{1.47608, 3.83841, 0.}, {1.74592, 5.58477, 0.}}	{25.1137, 45.3331, 108.}	inf	2945.	1639.73	{1253.24, 2145.73}	44.3214	0.262856	newly	3839.	3871.19	{3350., 4355.}	8.14613	0.0354659	Reff	1.99728	1.51613	{1.41307, 1.65635}	24.0904	ar	82690.	63071.	{56811., 71094.}	23.726
48	R0	{2.71771, 10.4636, 0.1}	{2.29245, 8.21152, 0.17}	{{1.98388, 6.25659, 0.}, {2.9884, 12.8326, 1.}}	{19.9126, 31.307, 242.}	inf	2061.	1515.98	{1031.34, 2313.87}	31.783	0.175307	newly	2437.	2264.44	{2020., 2497.}	8.84366	0.0409426	Reff	2.62862	2.22788	{1.93576, 2.88288}	19.3853	ar	91716.	84270.5	{79014., 93964.}	9.18969
49	R0	{2.44369, 6.66477, 0.1}	{1.71997, 3.62268, 0.04}	{{1.39162, 1.89883, 0.}, {1.94705, 4.66877, 0.}}	{29.616, 45.6444, 134.}	inf	1213.	607.048	{237.588, 899.617}	49.9548	0.351016	newly	3032.	2390.62	{1634., 3154.}	25.8681	0.146573	Reff	2.39345	1.69467	{1.37678, 1.91579}	29.1955	ar	88827.	68383.5	{50207., 78321.}	23.015
50	R0	{2.77383, 17.9775, 0.1}	{2.88692, 18.2261, 0.}	{{2.88523, 18.2127, 0.}, {2.88523, 18.2127, 0.}}	{4.07701, 1.38297, 100.}	inf	14883.	15629.1	{15390.8, 15796.7}	5.01296	0.0212078	newly	6948.	7315.36	{7200., 7430.}	5.28728	0.0223367	Reff	2.0682	2.13096	{2.12493, 2.13688}	3.03481	ar	92334.	93201.1	{93062., 93355.}	0.939047
